# Study on Black Spot Disease Detection and Pathogenic Process Visualization on Winter Jujubes Using Hyperspectral Imaging System

**DOI:** 10.3390/foods12030435

**Published:** 2023-01-17

**Authors:** Mengwei Jiang, Yiting Li, Jin Song, Zhenjie Wang, Li Zhang, Lijun Song, Bingyao Bai, Kang Tu, Weijie Lan, Leiqing Pan

**Affiliations:** 1 College of Food Science and Technology, Nanjing Agricultural University, Nanjing 210095, China; 2 College of Artificial Intelligence, Nanjing Agricultural University, Nanjing 210095, China; 3 College of Food Science and Technology, Hebei Normal University of Science & Technology, Qinghuangdao 066600, China; 4 College of Life Sciences, Tarim University, Alaer 843300, China; 5 Sanya Institute of Nanjing Agricultural University, Sanya 572024, China

**Keywords:** winter jujube, black spot disease, hyperspectral imaging, pathogenic process visualization

## Abstract

In this work, the potential of a hyperspectral imaging (HSI) system for the detection of black spot disease on winter jujubes infected by *Alternaria alternata* during postharvest storage was investigated. The HSI images were acquired using two systems in the visible and near-infrared (Vis-NIR, 400–1000 nm) and short-wave infrared (SWIR, 1000–2000 nm) spectral regions. Meanwhile, the change of physical (peel color, weight loss) and chemical parameters (soluble solids content, chlorophyll) and the microstructure of winter jujubes during the pathogenic process were measured. The results showed the spectral reflectance of jujubes in both the Vis-NIR and SWIR wavelength ranges presented an overall downtrend during the infection. Partial least squares discriminant models (PLS-DA) based on the HSI spectra in Vis-NIR and SWIR regions of jujubes both gave satisfactory discrimination accuracy for the disease detection, with classification rates of over 92.31% and 91.03%, respectively. Principal component analysis (PCA) was carried out on the HSI images of jujubes to visualize their infected areas during the pathogenic process. The first principal component of the HSI spectra in the Vis-NIR region could highlight the diseased areas of the infected jujubes. Consequently, Vis-NIR HSI and NIR HSI techniques had the potential to detect the black spot disease on winter jujubes during the postharvest storage, and the Vis-NIR HSI spectral information could visualize the diseased areas of jujubes during the pathogenic process.

## 1. Introduction

Winter jujube (*Ziziphus jujuba* Mill. cv. Dongzao) is considered one of the most important fresh-eating jujube varieties and is mainly cultivated in the north of China [1]. It is deeply appreciated by consumers for its favorable flavor and abundant nutrition components, such as phenolics, vitamins, amino acids, and numerous trace elements. [2,3]. However, winter jujubes are susceptible to postharvest diseases. Mainly, black spot disease, which is caused by *Alternaria alternata*, can cause fruit decay and quality reduction during storage, resulting in considerable losses to the winter jujube industry [4]. Therefore, it’s of great importance to realize the monitoring and detection of the black spot disease on winter jujubes. Moreover, it is reported that the black spot disease caused by *A. alternata* always occurs below the peel of the fruit with limited change in the early stages of infection [5]. Based on these facts, the disease can be rather difficult to detect with the naked eye before the visual damage becomes apparent. Developing a rapid and non-destructive method is thus urgently needed for the detection and discrimination of the black spot disease on winter jujubes.

Spectroscopic methods have been widely investigated in the quality detection of fruits, such as near infrared (NIR) spectroscopy, mid-infrared (MIR) spectroscopy, nuclear magnetic resonance (NMR), and fluorescence spectroscopy [6]. Among them, NIR spectroscopy is reported as one of the most advanced techniques for the detection of internal quality and defects in fruit, such as apples [7], oranges [8], kiwifruits [9], pomelos [10], and so on [11]. However, the point scanning on tested samples using NIR spectrometers only presents the characteristics of limited regions instead of the global overview of spatial information [12]. Hyperspectral imaging (HSI) technology can provide high resolution spatial information of objects compared with the conventional NIR spectroscopy, which enables the simultaneous acquisition of imaging and spectral information [13]. As a non-destructive detecting technology, HSI has been implemented in the quality (including sugar content, firmness, and so on) evaluations of fruits, such as apples [14,15,16], pears [17], etc. Since the presence of postharvest disease always affects the quality of fruits, HSI has also been used to detect the disease of fruit. Sun et al. [18] obtained an overall discrimination accuracy of 94.16% for fungal-infected peaches using a Vis-NIR (400–1000 nm) HSI system in the reflectance mode. Pan et al. [5] monitored the pathogenetic process and realized the early detection of the diseased pear with the overall accuracy of 97.5%. Moreover, a Vis-NIR hyperspectral system was established by Gomez-Sanchis et al. [19] to detect mandarin disease caused by *Penicillium digitatum*. In addition, the HSI has been applied for disease detection of apple [20], citrus [21], olive [22], and so on. However, to the best of our knowledge, limited studies provided insights into the disease detection and disease area visualization on winter jujubes during the process of infection using HSI techniques. Furthermore, most of these aforementioned works considered using an HSI system in the visible and near-infrared (Vis-NIR) region at 400–1000 nm for the disease detection of fruits. One of our interests in this work is to compare the discrimination ability of different HSI systems at Vis-NIR and short near-infrared region (SWIR) at 1000–2000 nm to detect the postharvest diseases on winter jujubes.

**Figure 1:**
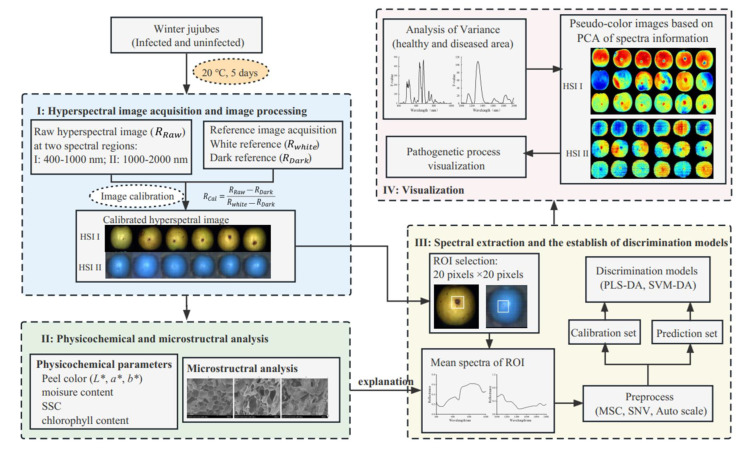
Procedures for black spot disease detection and pathogenetic process monitoring on winter jujube fruit using the HSI system in two spectral regions. (L*: lightness (0 = dark, 100 = light); a*: greenness (−)/redness (+); b*: blueness (−)/yellowness (+); SSC: soluble solids content; ROI: region of interest; PLS-DA: partial least squares discrimination analysis; SVM-DA: support vector machine discrimination analysis; PCA: principal component analysis).

In this study, the HSI images of winter jujubes infected by *A. alternata* in Vis-NIR (400–1000 nm) and SWIR (1000–2000 nm) spectral regions were both acquired during the postharvest storage of 5 days in order to: (i) investigate the changes of physical, chemical and structural properties on winter jujubes during the pathological process at the storage at 20 °C; (ii) explore the variations of spectral information on diseased jujubes and establish discrimination models based on the acquired spectral data; and (iii) highlight the disease tissues of winter jujubes at different infection stages (Figure 1).

## 2. Materials and Methods

### 2.1. Winter Jujube Samples

Fruit samples were harvested in Dali County, Shaanxi Province, on 25 October 2021 and purchased from a local fruit wholesale market in Nanjing, Jiangsu Province, on 26 October 2021. Around 9 kg of ‘Dali’ winter jujube fruits of similar maturity (at the mature green stage) [23] and shape, and without any visual defects, bruises, and visible diseases, were selected for the research. All samples were sterilized in sodium hypochlorite solution (2%, *v*/*v*) for 2 min, then rinsed with sterile water, and finally dried in air prior to use.

*Alternaria alternata*, preserved in the laboratory at the College of Food Science and Technology, Nanjing Agricultural University, was cultured on potato dextrose agar (PDA) medium at 28 °C and 95% relative humidity (RH) for seven days. The fungal spores were washed down and suspended in sterile water. In order to remove the mycelium, the above suspension was filtered through four layers of sterile gauze. The concentration of the fungal spore suspension was finally adjusted to 1 × 10^5^ CFU/mL via a hemacytometer.

In total, 240 samples were selected and labeled as the diseased group. A wound (2 mm wide by 2 mm deep) was made on the equator of each fruit using a sterile needle. Furthermore, 5 μL of the fungal spore suspension described above was injected into the wound to obtain the *A. alternata* inoculation samples. 240 samples with no treatment and 240 samples inoculated with an equivalent amount of sterile water were set up as two control groups to eliminate the influence of the natural maturation process and the mechanical injury caused by the inoculation process during storage. The samples were stored at 20 °C, 90% RH for five days. For each group, a total of 40 samples were taken for hyperspectral image acquisition at each sampling time (Day 0, 1, 2, 3, 4, and 5). After hyperspectral image acquisition, the samples were used for further detection (including physicochemical analysis and SEM analysis).

### 2.2. Physical, Chemical and Microstructural Characterizations

The color of the peel was measured near the inoculation position using a CR-10 portable colorimeter (Minolta co., Japan). The L*, a*, b* values were measured three times, and the average values were recorded for further analysis. The moisture content of jujube samples was determined by an oven-drying method according to the Chinese standard GB 5009.3–2016. In total, 2 g of flesh near inoculated position was collected and air-dried at 105 ± 5 °C until the constant weight was achieved, and the weight of jujubes before and after the drying treatment were measured to calculate the moisture content. SSC was determined using a PAL-1 refractometer (ATAGO, Tokyo, Japan) based on the juice that was obtained by squeezing and filtering the pulp. The chlorophyll content of the peel at the diseased area was detected by measuring the absorption at both 649 nm and 665 nm using a UV-1800 spectrophotometer (Shimadzu co., Tokyo, Japan), following the procedure of Sun et al. [18]. The physicochemical parameters mentioned above were also measured on the same positions of uninfected samples, which were adopted as contrasts.

In addition, the microstructures of the jujube flesh were observed using a scanning electron microscope (SEM; Hitachi SU8010, Tokyo, Japan). Before imaging, the flesh pieces were cut from the diseased area with a size of 4 mm × 4 mm × 2 mm and immersed in a 2.5% glutaraldehyde solution at 4 °C for 24 h. Furthermore, the samples were dehydrated in a graded series of ethanol solutions (30%, 50%, 70%, 90%, 95%, and 100%) for 20 min. After they were freeze-dried, the samples were sprayed with gold for 60 s to obtain the prepared specimens, following the procedure described by Sun et al. [24].

### 2.3. Hyperspectral Imaging System and Acquisition

Hyperspectral images of all samples in three groups were acquired using two lab-scale hyperspectral imaging (HSI) systems in reflectance mode. The visible and near-infrared (Vis-NIR) HSI system (I) (Isuzu Optics Co., Taiwan, China) consisted of an ICLB1620 CCD camera (Imperx Co., Boca Raton, FL, USA) and an imaging spectrometer (ImSpector V10E, Specim, Oulu, Finland), while the short-wave infrared (SWIR) HSI system (II) (Isuzu Optics Co., Taiwan, China) was comprised of a Raptor EM285CL camera (Raptor Photonics Co., Larne, UK) and an imaging spectrometer (ImSpector N25E, Specim, Oulu, Finland). 420 bands in the reflectance profiles in the range of 400–1000 nm with a spectral resolution of 2.8 nm and 143 bands over 1000–2000 nm with a spectral resolution of 6.5 nm were retained in the Vis-NIR and SWIR HSI systems, respectively. To acquire non-deformable and complete images and reduce the shadow area, the parameters were set after pretesting as follows: the lamps were fixed at an angle of 45 ° above the sample. The camera exposure time was 3 ms. The speed of the transport platform and the distance between the sample and camera were set to 7.23 mm/s and 28 cm for the Vis-NIR section and 6.2 mm/s and 24 cm for the SWIR section, respectively.

The acquired hyperspectral images should be corrected to reduce the influence of uneven illumination distribution and the dark current of the camera and eliminate redundant information. The original images were corrected according to Equation (1):

(1) $$R_{Cal}=\frac{R_{Raw}-R_{Dark}}{R_{white}-R_{Dark}}$$

where *R_Cal_* presents the corrected image. *R_Dark_* presents the dark reference image acquired with the camera covered, and *R_White_* presents the white reference image captured from a Teflon white board with 99.99% reflectivity.

### 2.4. Spectral Extraction and Processing

#### 2.4.1. Spectral Extraction

It is of crucial importance to select the region of interest (ROI), as the proper ROI can not only reflect the characteristics of the sample but also improve the performance of the model [25]. Thus, a square area (20 pixels × 20 pixels) centered on the inoculation sites, as well as the corresponding position of uninoculated samples, was defined as the region of interest. The extraction of spectral characteristics and the calculation of mean spectra values were performed via MATLAB R2019b (MathWorks. Inc, Natick, MA, USA).

#### 2.4.2. Spectral Processing

To reduce or eliminate useless information in the raw spectral data like noise, absorption peak overlap, baseline drift, and non-uniformity in samples and surfaces [26,27], three preprocessing methods were employed in this study, including standard normalized variate (SNV), multiplicative scatter correction (MSC), and auto scale. These methods could also enhance the effective spectral information and improve the accuracy and stability of the model. In this study, the PLS_Toolbox 7.5 (Eigenvector Research Inc., Wenatchee, WA, USA) in Matlab R2019b was applied to pretreat the spectra.

### 2.5. Statistic and Analysis

Partial least squares discriminant analysis (PLS-DA) is a classical linear classification modelling method. The general idea of PLS-DA is to estimate the linear regression between the input spectral information and the response class [28]. Support vector machine discriminant analysis (SVM-DA) is a nonlinear classifier based on supervised learning, which is always used for the analysis of hyperspectral data [29]. To compare the ability of different modelling methods for the discrimination of diseased jujubes at different stages of infection, two supervised classification methods (i.e., PLS-DA and SVM-DA) were carried out in the present study. Before the models were established, the entire dataset was randomly split into a calibration set and a prediction set in a 3:1 ratio. Eventually, 162 samples (27 samples × 6 class) were taken for the calibration set, while the remaining 78 samples (13 samples × 6 class) were taken for the prediction set. The models were developed using MATLAB R2019b, and the overall accuracy and per-class accuracy were used to evaluate the performance of the discrimination models.

The Shapiro-Wilk test was used to assess the normality of data distribution using IBM SPSS 20 (The SPSS Inc. Chicago, IL, USA). After that, the ANOVA analysis of physicochemical parameters and the spectra was performed using IBM SPSS 20. Principal component analysis (PCA) is a classical dimensionality reduction method which has been widely used. In this study, PCA was employed to realize the dimensionality reduction of spectral information in the range of both 400–1000 nm and 1000–2000 nm. The first three principal components (PC) of spectral information were extracted, and then the PC scores of each picture on the selected area of the hyperspectral image were calculated and displayed in different colors. PCA and pseudo-color images were performed using MATLAB R2019b.

## 3. Results and Discussion

### 3.1. Physical, Chemical and Microstructural Changes of Winter Jujubes

The data for all physicochemical parameters were normally distributed according to the results of the Shapiro-Wilk test. For the color parameters, the L* values of diseased jujubes showed a consistent decrease (*p* < 0.001) from 71.8 to 55.7 during the infection process (Table 1), and it declined more rapidly than the uninfected samples. The a* values presented a consistent increase trend during the infection and finally reached 5.5 at the end of the storage (Day 5), which was much higher (*p* < 0.001) than the a* value of uninfected groups from–0.3 to–0.4. These phenomena were in line with the previous results related to the differences in peel redness between healthy and diseased peaches [24]. For the infected jujubes, the variations of L* and a* values were not obvious (*p* > 0.05) at the early stage of infection at Days 0, 1, and 2, due to the latent nature of the pathogen [30]. Since Day 3, after inoculation, the jujube tissues gradually oxidized and the surface turned brown during the infection process, resulting in significant decreases in L* values and increases in a* values (*p* < 0.001). Comparatively, no significant changes (*p* > 0.05) of b* value were observed between the uninfected and infected groups.

Similarly, a slight decline (*p* < 0.05) of the chlorophyll content of jujubes could be observed during the first two days after inoculation, but there was a more intensive decrease (*p* < 0.001) after the third day of storage, ranging from 7.9 × 10^−2^ to 3.4 × 10^−2^ g/kg (Table 1). Although the chlorophyll content of uninfected jujubes could be decomposed during postharvest storage (Table 1), the infection of fruit would exacerbate the breakdown of chlorophyll. Different from the uninfected samples, SSC showed a significant decrease (*p* < 0.01) since Day 4 after inoculation with *A. alternata*, and the mean value of SSC was at a low level (14.3%) at the end of storage (Day 5). This was consistent with the previous results showing a rapid decomposition of sugar contents under the action of fungi [24]. The rapid depletion of sugars during infestation by pathogens also explained the reduction (*p* < 0.01) in the water content, which was an important substrate for chemical reactions.

**Figure 2:**
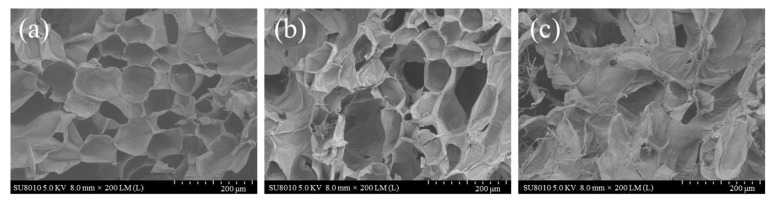
Scanning electron microscopy (SEM) of infected winter jujubes on (**a**) Day 0, (**b**) Day 2 and (**c**) Day 5 during the storage at 20 °C.

The changes in cell microstructure on infected winter jujubes during postharvest storage were observed by SEM and presented in Figure 2. The cells of jujube flesh were arranged in a neat and regular pattern with an intact structure at the beginning of storage (Day 0), and the cell walls were smooth and integrated (Figure 2a). After 2 days of storage, the cells exhibited the loose cell arrangement, and the cell cross-sectional area became larger owing to the swelling of the cells (Figure 2b). The structure of flesh cells was slightly shrunken, and a few broken cells could also be observed with some holes, suggesting that the cells had been affected by a fungal infestation. However, at the end of storage (Day 5), the structure of flesh tissue was completely destroyed (Figure 2c). The flesh cell walls exhibited severe shrinkage and deformation as the hyphae grew through the flesh cells of infected winter jujubes.

### 3.2. Spectral Characteristics of Winter Jujubes

#### 3.2.1. Variations of Spectra during the Infection

**Figure 3:**
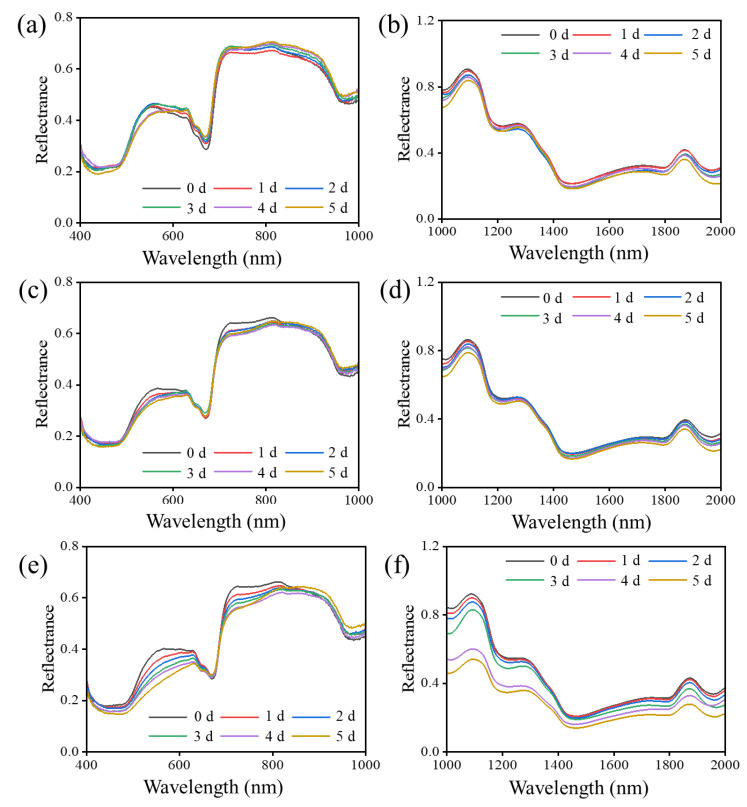
Mean spectra of control groups and infected groups during the storage at 20 °C in the Vis-NIR (400–1000 nm) and SWIR (1000–2000 nm) region. (**a**) Mean spectra of control group I (healthy samples without treatment) in the Vis-NIR region; (**b**) Mean spectra of control group I in the SWIR region; (**c**) Mean spectra of control group II (samples inoculated with sterile water) in the Vis-NIR region; (**d**) Mean spectra of control group II in the SWIR region; (**e**) Mean spectra of infected group (samples inoculated with *A. alternata*) in the Vis-NIR region; (**f**) Mean spectra of infected group in the SWIR region.

The averaged reflectance HSI spectra (Figure 3) in the Vis-NIR (400–1000 nm) and SWIR (1000–2000 nm) ranges were acquired based on the ROIs of jujubes in two control groups and inoculated jujubes at six different infection periods. According to Figure 3a–d, limited changes in spectra in both the Vis-NIR and SWIR regions of the two control groups were observed during storage. However, the mean reflectance spectra of infected winter jujubes presented an overall downward trend in the Vis-NIR region (400–1000 nm) during the 5 days of storage, as shown in Figure 3e. Similar downward trends were also observed at the whole tested wavelength range of 1000–2000 nm (Figure 3f). It was demonstrated that cell size and integrity of tissue would influence the light scattering [31]. More chances were provided for light to interfere with the cellular material, and light would be more absorbed and scattered when the cell structure of the flesh tissue was gradually damaged (Figure 2) [32]. Similar results were reported in the research on tomatoes [33]. Furthermore, the decrease of the lightness (Table 1) of the surface also attributed to the decreasing in both the Vis-NIR and SWIR regions. Particularly, the reflectance spectral curves of infected winter jujubes presented a significant decline in the region of 400–620 nm as storage days increased (Figure 3c). The possible reason to explain the phenomenon could be that the browning of the ROI area due to the oxidation of tissue led to the decline of the spectra since the valley around 500 nm was associated with pigments responsible for fruit color [34]. It was also in line with the results mentioned in Section 3.1 that the a* value increased during storage. The weakness of the absorption trough at around 670 nm was attributed to the reduction of the chlorophyll content [35] (Section 3.1). The peaks and valleys existed at 1180, 1320, and 1450 and were related to the absorption of water [36]. In summary, the changes in physical and chemical properties of the winter jujubes during the infection with *A. alternata* led to the spectra variations.

#### 3.2.2. Variations of Spectra between Healthy and Diseased Area

**Figure 4:**
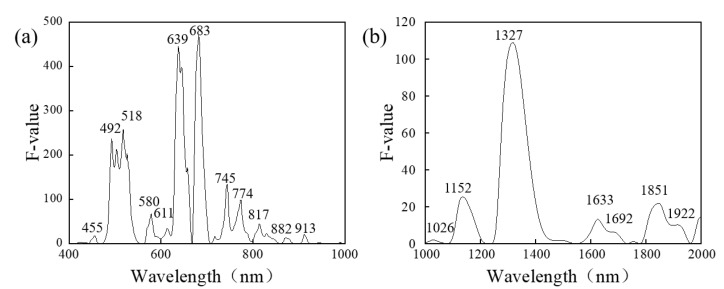
ANOVA results of SNV pre-treated spectra in the band range of (**a**) 400–1000 nm and (**b**) 1000–2000 nm.

A one-way ANOVA was performed between the SNV pretreated spectra in both the Vis-NIR (Figure 4a) and SWIR (Figure 4b) regions of the diseased and healthy areas based on diseased jujube samples at the end of storage (Day 5) to further explore the spectral variation of jujube samples after infection. In the Vis-NIR spectral region, the dominated spectral variations were mainly located at the wavelengths of 470–540 nm, 630–650 nm, 670–700 nm, and 730–780 nm (*p* < 0.05), as shown in Figure 4a. Particularly, the specific peaks at 492 nm, 518 nm, 639 nm, and 683 nm showed relatively high F-values (>200), suggesting the potential of these wavelengths to differentiate the diseased tissue from the sound tissue. The wavelengths at 492 nm and 518 nm were respectively related to carotenoid and total anthocyanin [37,38], which were both linked to the peel color such as a* value. While the informative bands at 639 nm and 683 nm represented the absorption of chlorophyll-*a* and *b* [5].

As for the SWIR spectral region, an ANOVA suggested major variations in 1120–1170 nm, 1260–1410 nm, 1600–1700 nm, and 1800–1940 nm (*p* < 0.05) (Figure 4b). Specifically, the wavelengths at 1152 nm, 1327 nm, and 1851 nm were noted with an F-value higher than 20. Among them, the wavelengths at 1152 nm and 1327 nm were near the strong carbohydrate absorbance bands [39]. While the wavelength at 1851 nm was related to the O-H stretching vibration of sugar [40], associated with SSC.

Interestingly, the variation of the spectra in the range of 400–1000 nm was shown to be higher (F-value of 500) (Figure 4a) than that of 1000–2000 nm (F-value of 120) (Figure 4b), which indicated the spectra in the Vis-NIR region were more suitable for the identification of diseased areas on winter jujubes during the infection process.

### 3.3. Supervised Classification Models

The PLS-DA and SVM-DA models were developed based on the raw and three different pretreated (MSC, SNV, and Auto scale) Vis-NIR and SWIR HSI spectra from the ROIs of all winter jujubes at different infection stages (Table 2). Generally, the discrimination accuracy of the prediction models based on the MSC and SNV pretreated HSI spectra in both the Vis-NIR and SWIR regions was higher than those using the raw spectra without preprocessing. Among these pre-processing methods, SNV treatment was the best pre-processing method for both the Vis-NIR and SWIR spectrums, with the highest discrimination accuracy over 88.46% and 87.18% of the prediction dataset, respectively. Based on that, the SNV pre-processing strategy was further considered to discriminate the different infection processes of jujube samples (Table 3).

As presented in Table 3, the PLS-DA model provided a better classification performance than the SVM-DA model for spectra in the Vis-NIR range, with an overall accuracy of 95.68% and 92.31% for the calibration and prediction sets, respectively, while the SVM-DA model performed a poor discrimination (acc = 88.46%). For the discrimination models based on SWIR spectra, PLS-DA models showed better overall classification performances for both the calibration (acc = 93.21%) and prediction set (acc = 91.03%) compared with the SVM-DA model. However, these two SWIR discrimination models gave a poor classification for the prediction set on Day 2 after inoculation, with accuracy values ranging from 61.54% to 69.23%. This could be attributed to the slight changes in symptoms and chemical components at the early stages of infection, which resulted in the misclassification of the samples into other infection periods. Similar results could be found in the previous study of pears during the *A. alternata* infection periods [5]. Particularly, a more satisfactory classification accuracy (acc = 92.31%) at the end of the storage (Day 5) was observed based on the SWIR models, which was relatively higher than the Vis-NIR models (acc = 84.62%). The possible explanation was the infected samples at the end of storage (Day 4 and 5) could be easily misclassified based on the visible spectral region presenting the similar symptoms (black spot on the skin) of the infected samples.

Interestingly, the overall acc values in the Vis-NIR spectral region were relatively higher than those in the SWIR spectral region, indicating that spectral bands at 400–1000 nm had a better ability to detect the postharvest infection of winter jujubes than the bands at 1000–2000 nm. This could be due to the fact that the Vis-NIR region can present both the absorption bands of a large variation of jujube pigments and some biochemical information, while the SWIR spectral region is more sensitive to the variation in chemical components during the postharvest infection. Actually, the appearances and physical properties of winter jujubes varied more than their chemical attributes, as seen from the spectroscopic point of view (described in Section 3.2.2). Moreover, a similar phenomenon can be found in previous studies for quality and defect detection on jujubes when using the HSI system over a broad spectral region covering both Vis-NIR and SWIR [41,42]. Consequently, the SNV-PLS-DA models based on Vis-NIR and SWIR have the potential to identify and discriminate the diseased winter jujubes, and the HSI spectral information in the Vis-NIR region showed a better discrimination performance than the SWIR region.

### 3.4. Visualizing the Pathogenetic Process of Jujubes

**Figure 5:**
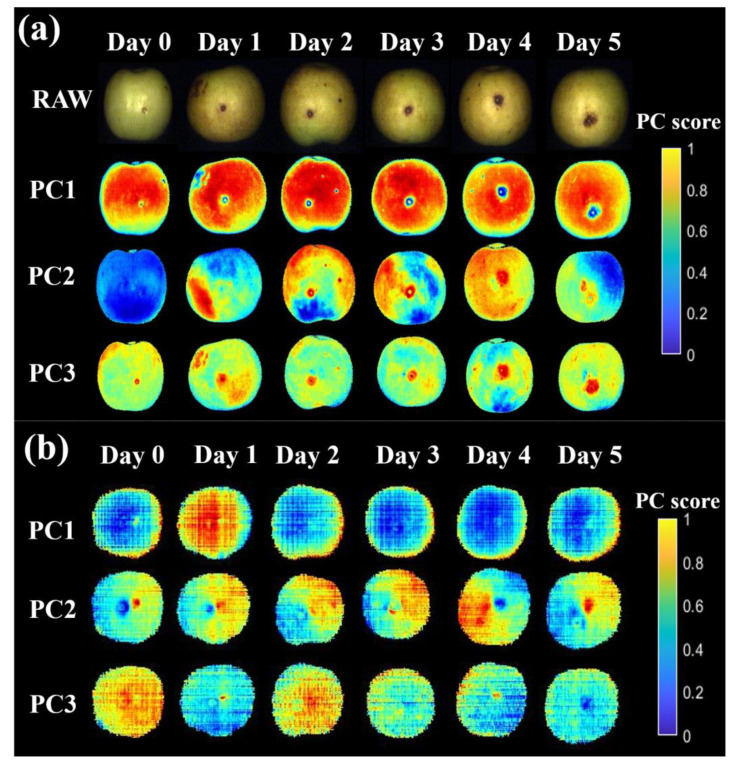
Raw and pseudo-color images based on PC scores of the inoculated fruit at different infection stages in the range of (**a**) 400–1000 nm and (**b**) 1000–2000 nm.

In this part, PCA was performed on the whole Vis-NIR (Figure 5a) or SWIR (Figure 5b) HSI spectra of jujubes samples, in order to highlight the diseased areas from the sound tissues at different infection stages. The photography and pseudo-color images based on the first three PC scores of two HSI systems were displayed in Figure 5, relating to the same inoculated jujube at different infection stages.

**Figure 6:**
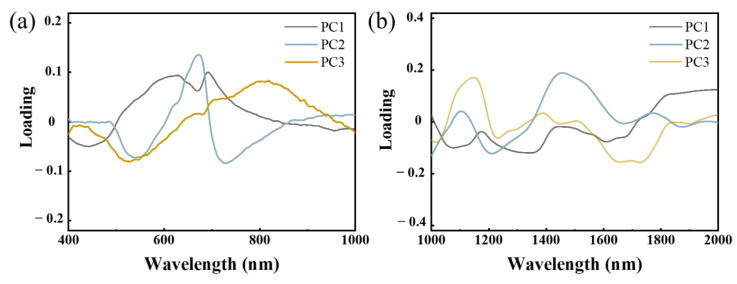
The loading plots of PC1, PC2 and PC3 in the range of (**a**) 400–1000 nm and (**b**) 1000–2000 nm.

For the images acquired by the Vis-NIR HSI system, the infected areas on the surface of winter jujubes were difficult to detect at the early stage of infection based on the photography (Figure 5a). While visual symptoms could be clearly observed from the third day after inoculation, which was in line with their obvious physicochemical changes, as described by the results in Section 3.1. The PC1 images of winter jujubes clearly highlight the diseased areas of jujubes at the different infection stages, with the explanation rate over 14.51% (Figure 5a). Compared with the PC1 images, the PC2 images showed poor performance on differentiating diseased areas, as the color distribution was irregular. Although PC3 images could roughly distinguish diseased areas from healthy areas, mechanical wounds made prior to inoculation were misjudged as diseased areas, which was improper. The explanation rates of PC2 and PC3 ranged from 3.74% to 9.10% and 2.13% to 3.14%, respectively. Based on the PC1 images, there was a small infected area marked with a blue color that could be observed on the first two days (Days 1 and 2), and the infected areas became more noticeable at the end of storage (Days 4 and 5), while the red and yellow areas were noted as healthy tissue. The loading plot (Figure 6a) showed the relative contribution of spectra in the whole Vis-NIR spectral range for PC1. The most contributed wavelengths of PC1 images to discriminate the diseased areas of jujubes were located at 440 nm, 638 nm, 670 nm, 695 nm, and 950 nm (Figure 6a). Interestingly, there was an overlap between the crucial wavelengths obtained according to the PCA analysis and those extracted based on the result of the ANOVA (Section 3.2.2). Specifically, the wavelengths at 638 nm, 670 nm, and 695 nm were associated with the chlorophyll content, which was in line with the previous findings that the chlorophyll content would be significantly affected by pathogen infection [18].

However, for the images acquired by the SWIR HSI system, it seemed difficult to identify the diseased area on the jujube samples (Figure 5b). The possible reason was the limited variation of the spectra between the healthy and diseased areas in the near-infrared region (Section 3.2.2), which resulted in similar PC scores of spectra for each picture. This was also consistent with the results obtained in Section 3.3, which showed that the spectra in the range of 400–1000 nm were more informative than those in 1000–2000 nm. Therefore, the PC1 score images based on the spectra in the Vis-NIR region (400–1000 nm) could highlight the diseased areas of jujubes during the pathogenetic process.

## 4. Conclusions

The research was conducted to explore the feasibility of applying HSI for the detection of black spot disease on winter jujubes, and the performance of spectral information in Vis-NIR (400–1000 nm) and SWIR (1000–2000 nm) regions was compared. The physical, chemical, and microstructural properties were also characterized during the pathogenic process. The variations of these properties affected the spectral profile in both the Vis-NIR and SWIR regions, resulting in an overall downward trend during the infection process. Three pre-processing methods and two classification models were employed to discriminate the diseased jujubes at different infection stages. Among them, SNV-PLS-DA based on both Vis-NIR and SWIR spectra yielded the most satisfying performance, with an overall accuracy of 92.31% and 91.03%, respectively. Moreover, the diseased area could be accurately distinguished from sound tissue using PCA on the spectra in the Vis-NIR region, while poor performance was received when it came to the SWIR region. These results revealed the potential of HSI for the detection of black spot disease on winter jujubes, and the spectra in Vis-NIR (400–1000 nm) region were proved more suitable for the diseased detection and disease process visualization on winter jujubes. The findings of this study provide useful ideas and references for the development of disease detection methods for fruit based on hyperspectral imaging technology.

## Figures and Tables

**Table 1:** Physical and chemical parameters of uninfected and infected winter jujubes during the storage at 20 °C.

Group	Storage Periods	L*	a*	b*	Moisture Content	SSC (%)	Chlorophyll (×10^−2^ g/kg)
Control group I (Healthy)	Day 0	71.2 ± 0.4 ^a^	−3.3 ± 0.9 ^a^	41.0 ± 0.6 ^a^	85.4 ± 0.8 ^a^	15.6 ± 0.1 ^a^	8.7 ± 0.3 ^a^
	Day 1	70.3 ± 1.0 ^ab^	−2.5 ± 0.4 ^a^	41.1 ± 1.0 ^a^	85.2 ± 0.9 ^a^	15.7 ± 0.3 ^a^	8.7 ± 0.2 ^a^
	Day 2	69.6 ± 1.3 ^abc^	−1.7 ± 0.7 ^ab^	40.7 ± 0.8 ^a^	85.0 ± 1.0 ^ab^	15.5 ± 0.2 ^a^	8.4 ± 0.2 ^ab^
	Day 3	68.2 ± 1.0 ^bcd^	−1.4 ± 0.6 ^ab^	41.0 ± 1.0 ^a^	84.7 ± 0.4 ^ab^	15.4 ± 0.3 ^a^	8.1 ± 0.4 ^ab^
	Day 4	67.7 ± 1.0 ^cd^	−0.9 ± 0.8 ^bc^	40.0 ± 0.5 ^a^	84.4 ± 0.7 ^ab^	15.4 ± 0.1 ^a^	7.7 ± 0.4 ^b^
	Day 5	66.5 ± 1.7 ^d^	−0.4 ± 0.5 ^c^	40.1 ± 0.7 ^a^	83.7 ± 0.6 ^c^	15.3 ± 0.2 ^a^	6.7 ± 0.4 ^c^
Control group II (Inoculated with Sterile water)	Day 0	70.7 ± 0.5 ^a^	−3.3 ± 0.9 ^a^	41.4 ± 0.7 ^a^	85.3 ± 0.7 ^a^	15.7 ± 0.3 ^a^	8.8 ± 0.2 ^a^
	Day 1	70.4 ± 1.0 ^a^	−2.0 ± 0.3 ^a^	40.7 ± 1.0 ^a^	85.0 ± 0.4 ^ab^	15.7 ± 0.1 ^a^	8.6 ± 0.3 ^a^
	Day 2	68.1 ± 1.0 ^b^	−1.4 ± 1.3 ^a^	40.5 ± 1.0 ^a^	84.8 ± 0.8 ^ab^	15.4 ± 0.3 ^a^	8.2 ± 0.2 ^ab^
	Day 3	67.4 ± 1.0 ^bc^	−1.0 ± 1.0 ^ab^	41.1 ± 0.3 ^a^	84.4 ± 0.7 ^ab^	15.5 ± 0.1 ^a^	7.8 ± 0.4 ^bc^
	Day 4	66.9 ± 1.1 ^bc^	−1.0 ± 1.2 ^ab^	40.7 ± 0.9 ^a^	84.2 ± 0.4 ^b^	15.4 ± 0.2 ^a^	7.1 ± 0.4 ^cd^
	Day 5	65.4 ± 1.3 ^c^	−0.3 ± 0.8 ^b^	40.3 ± 1.0 ^a^	83.1 ± 0.5 ^c^	15.2 ± 0.4 ^a^	6.5 ± 0.3 ^d^
Infected group (Inoculated with *A. alternata*)	Day 0	71.8 ± 0.7 ^a^	−3.2 ± 1.1 ^a^	41.4 ± 1.0 ^a^	85.6 ± 0.9 ^a^	15.7 ± 0.2 ^a^	8.8 ± 0.2 ^a^
	Day 1	69.2 ± 1.0 ^b^	−2.1 ± 0.7 ^b^	41.1 ± 0.6 ^a^	84.6 ± 0.8 ^a^	15.5 ± 0.2 ^a^	8.7 ± 0.3 ^ab^
	Day 2	67.9 ± 0.4 ^b^	−0.8 ± 1.1 ^b^	41.5 ± 1.2 ^a^	84.3 ± 0.6 ^a^	15.4 ± 0.1 ^a^	7.9 ± 0.1 ^b^
	Day 3	61.7 ± 0.6 ^c^	2.4 ± 1.2 ^c^	41.5 ± 1.2 ^a^	82.8 ± 0.9 ^b^	15.2 ± 0.2 ^a^	6.7 ± 0.1 ^c^
	Day 4	57.0 ± 1.2 ^d^	3.3 ± 1.0 ^cd^	42.2 ± 1.2 ^a^	81.7 ± 0.9 ^bc^	14.7 ± 1.6 ^b^	4.9 ± 0.5 ^d^
	Day 5	55.7 ± 0.9 ^d^	5.5 ± 0.8 ^d^	40.0 ± 0.5 ^a^	81.2 ± 0.7 ^c^	14.3 ± 0.3 ^b^	3.4 ± 0.6 ^e^

L*: lightness (0 = dark, 100 = light); a*: greenness (−)/redness (+); b*: blueness (−)/yellowness (+); Different letters on the same columns denote a significant difference (*p* < 0.05).

**Table 2:** Comparison of PLS-DA and SVM-DA models based on the raw, MSC, SNV and Auto scale spectral preprocessing methods of Vis-NIR (400–1000 nm) and SWIR (1000–2000 nm) wavelengths.

Overall Accuracy (%)		Vis-NIR (400–1000 nm)		SWIR (1000–2000 nm)	
		PLS-DA	SVM-DA	PLS-DA	SVM-DA
Raw	Cal.	95.06	93.83	84.57	84.57
	Pre.	89.74	80.77	73.08	73.08
MSC	Cal.	96.30	91.98	90.74	91.98
	Pre.	91.02	88.46	88.46	87.18
SNV	Cal.	95.68	93.83	93.21	92.59
	Pre.	92.31	88.46	91.03	87.18
Auto scale	Cal.	95.06	94.44	90.12	78.40
	Pre.	88.46	81.62	85.90	70.51

Calibration set (Cal.) contained the 162 (27 samples × 6 class) infected samples at six infected stages; Prediction set (Pre.) contained the 78 (13 samples × 6 class) infected samples at six infected stages.

**Table 3:** Result of classification models based on SNV pre-treated HSI spectral in Vis-NIR (400–1000 nm) and SWIR (1000–2000 nm) region.

Overall Accuracy (%)	400–1000 nm				1000–2000 nm			
	PLS-DA		SVM-DA		PLS-DA		SVM-DA	
	Cal.	Pre.	Cal.	Pre.	Cal.	Pre.	Cal.	Pre.
Day 0	100.00	100.00	100.00	100.00	96.30	84.62	100.00	92.31
Day 1	96.30	92.31	96.30	84.62	96.30	100.00	88.89	92.31
Day 2	100.00	92.31	100.00	92.31	77.78	69.23	74.07	61.54
Day 3	100.00	92.31	92.59	84.62	92.59	100.00	100.00	100.00
Day 4	85.19	92.31	85.19	84.62	100.00	92.31	96.30	92.31
Day 5	92.59	84.62	88.89	84.62	96.30	100.00	96.30	84.62
Overall	95.68	92.31	93.83	88.46	93.21	91.03	92.59	87.18

Calibration set (Cal.) contained the 162 (27 samples × 6 class) infected samples at six infected stages; Prediction set (Pre.) contained the 78 (13 samples × 6 class) infected samples at six infected stages.

## Data Availability

Not applicable.
